# The effect of an intensive smoking cessation intervention on disease activity in patients with rheumatoid arthritis: study protocol for a randomised controlled trial

**DOI:** 10.1186/s13063-017-2309-5

**Published:** 2017-11-28

**Authors:** Ida Kristiane Roelsgaard, Thordis Thomsen, Mikkel Østergaard, Robin Christensen, Merete Lund Hetland, Søren Jacobsen, Lena Andersen, Hanne Tønnesen, Silvia Rollefstad, Anne Grete Semb, Bente Appel Esbensen

**Affiliations:** 1 Copenhagen Center for Arthritis Research (COPECARE), Center for Rheumatology and Spine Diseases, Rigshospitalet, Glostrup, Denmark; 2 Abdominal Centre, Rigshospitalet, Copenhagen, Denmark; 3 Department of Clinical Medicine, Faculty of Health and Medical Sciences, University of Copenhagen, Copenhagen, Denmark; 4 Musculoskeletal Statistics Unit, The Parker Institute, Bispebjerg and Frederiksberg Hospital, Copenhagen, Denmark; 5 DANBIO Registry, Center for Rheumatology and Spine Diseases, Rigshospitalet, Glostrup, Denmark; 6 Center for Rheumatology and Spine Diseases, Rigshospitalet, Copenhagen, Denmark; 7 WHO-CC, Bispebjerg-Frederiksberg Hospital, Copenhagen University, Copenhagen, Denmark; 8 Clinical Health Promotion Centre, Health Sciences, Faculty of Medicine, Lund University, Lund, Sweden; 9 Health, Faculty of Medicine, University of Southern Denmark, Odense, Denmark; 10 Preventive Cardio-Rheuma Clinic, Department of Rheumatology, Diakonhjemmet Hospital, Oslo, Norway; 11 The Danish Rheumatism Association, Gentofte, Denmark

**Keywords:** Motivational counselling, Nicotine replacement therapy, Cardiovascular disease, Behavioural change, Clinical health promotion, Smoking cessation intervention, Health-related quality of life, Tobacco smoking, Flare, Cardiovascular biomarkers

## Abstract

**Background:**

Rheumatoid arthritis (RA) is a chronic, inflammatory rheumatic disease with the potential to induce significant disability. Patients with RA are at increased risk of cardiovascular diseases (CVD). Smokers with RA tend to experience more pain and fatigue, higher disease activity, more erosive joint destruction and a lower health-related quality of life (HR-QoL) than non-smokers. It remains to be determined whether these effects can be reduced by smoking cessation.

This randomised controlled trial (RCT) in patients with RA aims to examine the effect of intensive smoking cessation intervention (motivational counselling combined with tailored nicotine replacement therapy) versus standard care on smoking cessation, and consequently on disease activity. Secondary objectives are to explore the effect on flare, risk factors for CVD, lung function, physical function, HR-QoL, pain and fatigue in patients with RA.

**Methods:**

This will be a multicentre, open label, two arm, parallel group, RCT, including 150 daily smokers with RA, being in remission or having low-moderate disease activity (DAS28 ≤ 5.1). The intervention group (n = 75) will receive five counselling sessions with a trained smoking cessation counsellor based on the principles of motivational counselling. Furthermore, intervention patients will be offered nicotine replacement therapy tailored to individual needs. Participants randomised to the control group will receive standard care. The co-primary outcome is a hierarchical endpoint, which will be evaluated at 3 months follow-up and will include (1) self-reported smoking cessation biochemically validated by exhaled carbon monoxide and (2) achievement of EULAR clinical response (an improvement in DAS28 of > 0.6). Follow-up visits will be performed at 3, 6 and 12 months post-intervention.

**Discussion:**

This trial will reveal whether intensive smoking cessation counselling helps smokers with RA to achieve continuous smoking cessation and whether, as a concomitant benefit, it will reduce their RA disease activity. The trial aims to generate high quality evidence for the feasibility of a health promotion intervention for smokers with RA.

**Trial registration:**

ClinicalTrials.gov, identifier: NCT02901886. Registered on 10 September 2016. Recruitment status updated on 10th October 2016.

**Electronic supplementary material:**

The online version of this article (doi:10.1186/s13063-017-2309-5) contains supplementary material, which is available to authorized users.

## Background

Rheumatoid arthritis (RA) is a chronic inflammatory disease with a prevalence of 1% in the general population [1]. The onset of RA occurs at all ages, but most frequently in women aged 50–60 years [2]. The disease is not curable per se; however, its course can be improved significantly by use of disease-modifying anti-rheumatic drugs [3, 4]. All joints may be affected, but symptoms are most common in hands and feet. During periods of high disease activity, patients can experience severe pain and fatigue, which may restrict social life as well as physical and daily life activities [4, 5]. Furthermore, patients with RA have a reduced health-related quality of life (HR-QoL) [6, 7] and are at increased risk of cardiovascular diseases (CVD), with a risk comparable to that of patients with diabetes mellitus [8]. There is evidence that patients with RA have a 1.6-fold higher rate of acute myocardial infarction and ischemic stroke than patients without RA [9]. The inflammatory process in RA appears to be linked to the increased risk of CVD [10].

Smoking is more prevalent among Danish patients with RA than in the general population. A recent survey reported that 30% of patients with RA were daily smokers [11] compared to 17% in the general Danish population [12]. Tobacco smoking is a well-known environmental risk factor for the development of RA and the association is well established [5, 10, 13–16]. Furthermore, smoking is associated with chronic persistent RA [17]. Several studies suggest that smoking may augment symptoms associated with RA. Smokers with RA have a tendency to experience more pain and fatigue, higher disease activity, swifter radiographic progression and reduced HRQoL than non-smokers with RA [18–21].

Several strategies are available to support smoking cessation. A Cochrane review indicated that smoking cessation based on individual behavioural counselling combined with nicotine replacement therapy (NRT) is the most effective intervention for long-term smoking cessation in the general population [22]. The process of behavioural change is a central part of smoking cessation [23], and includes different stages as described in the Stages of Change Model [24]. Smokers embarking on smoking cessation will often move back and forth between various stages [25], which often last several years. Motivational interviewing has proven to be essential in behavioural counselling, and has assisted healthy people and those with chronic diseases to quit smoking [26–28]. Cessation counselling related to smoking, incorporating the principles of motivational interviewing, aims to encourage smokers to reflect on the pros and cons of smoking cessation and their motivation for, and self-efficacy in regard to, quitting this habit [29]. There is scarce evidence for the effect of smoking cessation interventions for patients with RA [30, 31]. In a non-controlled intervention study, 55 patients with RA received brief smoking cessation advice (3–5 minutes) from a rheumatologist followed by 20 minutes of verbal and written smoking cessation advice from a nurse [31]. Patients were additionally offered undefined pharmacological therapy. At 3 months follow-up, self-reported sustained smoking quit rates were 11.8%, indicating that smoking cessation among patients with inflammatory joint diseases may be achievable. The intervention that had the highest rate of smoking cessation was the combination of behavioural intervention and NRT [22]; however, this type of intervention has not been tested in a randomised controlled trial (RCT) among RA patients. Hence, an RCT to test such an approach versus standard care is urgently required for these patients.

Our hypothesis is that an intensive smoking cessation intervention will contribute to smoking cessation in patients with RA, which will then mediate a reduction in disease activity.

### Aim

This RCT in patients with RA aims to examine the effect of intensive smoking cessation intervention (motivational counselling combined with tailored NRT) versus standard care on smoking cessation, and consequently on disease activity. Secondary objectives are to explore the effect on flare, risk factors for CVD, lung function, physical function, HR-QoL, pain and fatigue in patients with RA.

## Methods/Design

### Trial design

The study is an international, multicentre, randomised trial in which daily smokers with RA in remission or with low-moderate disease activity ≤ 5.1 DAS28 (Disease Activity Score based on 28 joints assessment, serum-C-reactive protein (CRP) and patient’s Global assessment of a visual analogue scale (VAS)) will be randomised 1:1 to either an intervention group or to a control group. Patients will be followed for 58 weeks, including the 6-week intervention period and at 3, 6 and 12 months into the post-intervention follow-up period.

### Study sites

We will recruit patients from the Center for Rheumatology and Spine Diseases, Rigshospitalet, Denmark, and from the Preventive Cardio-Rheuma Clinic, Department of Rheumatology, Diakonhjemmet Hospital, Oslo, Norway.

### Characteristics of participants

#### Inclusion criteria

Patients will be included in the study if they have RA as defined by the American College of Rheumatology 1987 criteria and/or European League Against Rheumatism (EULAR) 2010 criteria [32, 33], are over 18 years of age, smoking tobacco daily, and are able to understand and speak Danish or Norwegian, respectively. Furthermore, for the 3 months prior to inclusion patients need to have been in clinical remission or low-moderate disease activity (DAS28 ≤ 5.1) and in stable anti-rheumatic medical treatment as documented in (1) the DANBIO registry in Denmark, or (2) the electronic patient journal, Department of Rheumatology, Diakonhjemmet Hospital, Oslo, Norway.

#### Exclusion criteria

Patients will be excluded from the study if they have had a change of dose or preparation in anti-rheumatic medical treatment within the previous 3 months, or a scheduled change in anti-rheumatic medical treatment, including glucocorticoid injection during the previous month, are cognitively or otherwise unable to give informed consent, are pregnant or breastfeeding.

### Recruitment, screening and enrolment

Screening for potentially eligible patients at each trial site will be carried out as follows:

At the Danish sites, potential participants will be recruited by one of the following methods:

1. The DANBIO database, a nationwide registry including patients with inflammatory joint diseases in a longitudinal observational cohort [34], will be used to identify potential patients. Potential patients will be identified by using the search terms “rheumatoid arthritis” AND “smoker” (Fig. 1). Potentially eligible patients will receive an invitation to participate in the trial in a letter including written information about the trial and information about how the patient was identified in DANBIO. Three to four days after receiving the letter, the patient will be contacted by phone by the project manager (IKR) and invited to attend a meeting with a trained smoking cessation counsellor at which the trial will be further explained.

**Fig. 1:**
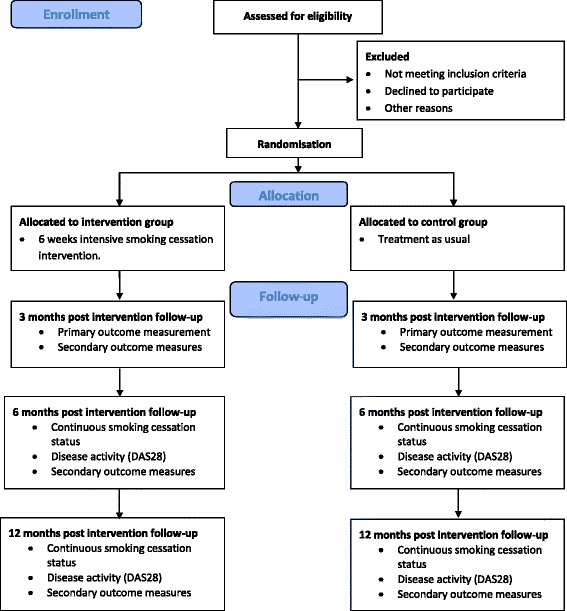
CONSORT Flow diagram: recruitment, screening and enrolment
2. At routine visits to the rheumatology outpatient clinic. Potentially eligible patients will be orally informed about the trial and will subsequently receive written participant information.
3. By referral from a physician or nurse from the rheumatology outpatient clinic. Potentially eligible patients will be orally informed about the trial and will subsequently receive written participant information.

The written participant information will contain information about the trial, background, objectives, course, measurements, and potential advantages and disadvantages for the patient. Furthermore, potential risks and adverse effects will be presented, and it will be stressed that participation is voluntary. Written informed consent will be obtained from each patient prior to inclusion in the trial.

At the Norwegian site, potential participants will be recruited as follows:

- At routine visits to the Preventive Cardio-Rheuma Clinic, Department of Rheumatology, Diakonhjemmet Hospital, Oslo, Norway. The information process will follow the same procedure as in Denmark.

Excluded patients, and eligible patients who do not want to participate, will be registered as either (1) not meeting the inclusion criteria, (2) refused to participate, or (3) excluded for other reasons.

### Power sample size considerations

#### Smoking cessation

The primary endpoint is a hierarchical outcome, hence an effect of ‘randomisation to smoking cessation intervention’ on disease activity will be statistically supported only if there is a statistically significant difference in the number of participants who quit smoking. We expect (conservatively) that 50% of participants in the intervention group will achieve continuous smoking cessation 3 months post-intervention (primary outcome 1a) versus 10% of participants in the control group [35–37], thus corresponding to a number-needed-to-treat of 1/(0.50 – 0.10) = 1/0.40 = 2.5 ≈ 3 patients. These proportions lead to a statistical power of 90% with a total sample size of 52, assuming a balanced design. Thus, even with 52 participants (26 in each group) the trial will have great statistical strength (90%) for the smoking cessation outcome.

#### Disease activity

For a comparison of two independent binomial proportions using Pearson’s χ^2^ statistic with a two-sided significance level of 0.05, a sample size of 65 participants per group will provide a power of at least 90% to detect a difference between the treatment groups, given an expected response rate (DAS28 improvement > 0.6) of 40% in the intervention group versus 15% in the control group [38, 39]. This corresponds to a number-needed-to-treat of 1/(0.40 – 0.15) = 1/0.25 = 4 patients.

As some attrition is expected it was decided to include a total of 150 participants in the trial, randomised 1:1. If inclusion of 150 participants cannot be reached within the given timeframe, 98 participants will be sufficient to achieve a statistical power of 80%.

### Randomisation and blinding

Immediately after collecting baseline data using stratified block randomisation (block size 6–10) participants will be randomised to either (1) the intervention group or (2) the control group. The stratification variables are trial site and anti-cyclic citrullinated peptide (anti-CCP status). The allocation sequence is generated using computer-generated random numbers. Participants will be informed about their group allocation directly after randomisation. For participants randomised to the smoking cessation intervention group, the first intervention meeting will be scheduled as soon as possible, preferably immediately after randomisation. It is not possible either to blind participants to their allocated intervention or to blind the project nurses performing the intervention. The primary outcomes will be assessed by blinded assessors and smoking cessation will be self-reported by participants and validated biochemically.

### Intervention and control group

#### Intervention group

The intervention includes (1) individual motivational counselling in combination with (2) tailored NRT.

1. Individual motivational counselling
  The intervention consists of five individual motivational counselling sessions, each lasting 20–40 minutes over a period of 6 weeks with a trained smoking cessation counsellor. The principles of motivational counselling are based on the transtheoretical model of change [24]. The smoking cessation counsellor has also been trained in motivational counselling techniques specific to this intervention. Six registered nurses from the participating centres will provide the intervention. The Danish nurses have all completed the same Danish Cancer Society training programme based on the motivational counselling approach. The Norwegian nurses are specialised in pulmonary diseases and use the same counselling approach taught at the Danish Cancer Society.
  The smoking cessation counsellor’s role is to ask, listen and follow the participants cue and to adapt formal information to the participant’s motivational stage. Two tools, the ‘Line’ and the ‘Box’, will be used in the intervention. The Line is a simple tool to open the dialogue. It is constructed as a VAS scale and involves two questions which concern (1) the importance of smoking cessation and (2) the participant’s own belief in their capability of stopping smoking [40]. The Box consists of four empty squares, which the participant is asked to fill out. The Box helps the participant reflect and support the lifestyle change [40].
  Each meeting contains different themes. The first meeting is an introduction to the counselling course and preparation for smoking cessation, including the participant’s smoking status and their motivation for cessation. The second meeting aims to prepare the participant for the three first days without smoking. The third meeting aims to help the participant with issues concerning quitting smoking, including risk situations, relapse, reward and social network, and smoking cessation. The fourth meeting includes maintaining motivation, physical activity, handling of stress and mood swings. The fifth (final) meeting includes continuing help with smoking cessation and preparation for the time after the intervention.
2. NRT
  The participants in the intervention group will be offered NRT free of charge and, if accepted, it will be tailored individually according to the Fagerström Test for Nicotine Dependence [41]. Participants will be able to choose between the NRT products, including a patch, chewing gum, inhalator or mouth spray. The participants will note their tobacco and nicotine replacement consumption in a smoking diary.

#### Control group – standard care

The control group will receive the standard treatment and care in the rheumatology outpatient clinic. Participants will be encouraged to write a diary describing their tobacco use during the trial period. If participants in the control group express an interest in receiving smoking cessation counselling, they will be informed about municipal programmes.

#### Outcome measures

The following outcomes will be assessed by blinded assessors:

1. Primary outcome: smoking status and DAS28 at 3 months follow-up. We will use the strictest definition of smoking cessation, i.e. self-reported continuous cessation, verified by exhaled carbon monoxide.
2. Arterial stiffness, serum lipids (total cholesterol, high- and low-density lipoprotein cholesterol, triglycerides) and glycated haemoglobin, blood pressure, pulse, waist circumference, body weight, height, lung function by forced expiratory volume in the first second.

The outcomes of smoking status, FLARE instrument, physical function by Health Assessment Questionnaire (HAQ), HR-QoL by SF-36 and EuroQoL 5D, pain by VAS and fatigue by the Bristol Rheumatoid Arthritis Fatigue Numeric Rating Scale (BRAF-NRS), will be self-reported.

### Primary outcome measure

**Fig. 2:**
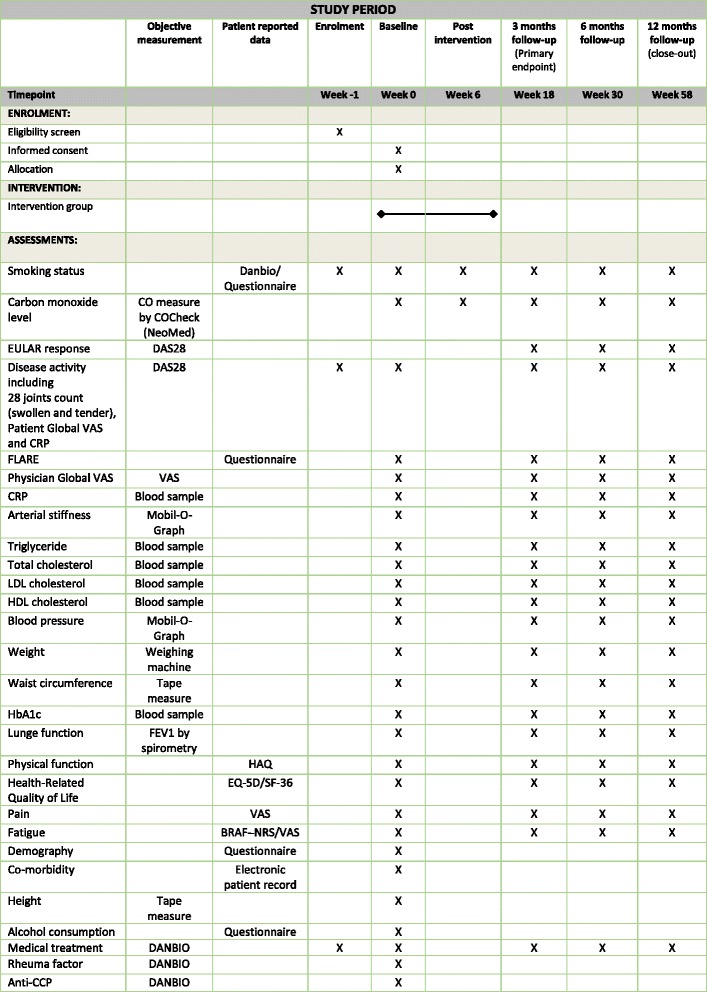
SPIRIT figure

The primary endpoint will be measured at 3 months follow-up (Fig. 2). It is a hierarchical endpoint comprising (1a) self-reported continuous smoking cessation validated by exhaled carbon monoxide at 3 months follow-up and (1b) EULAR clinical response (an improvement in DAS28 of > 0.6) at 3 months follow-up.

Endpoint 1b will be tested only if we identify a statistically significant increase in the number of intervention participants achieving continuous smoking cessation at 3 months follow-up. The participants will need to participate in all five meetings in the intervention in order to complete it.

- Smoking status
  The participants’ smoking status will be monitored by self-reported continuous smoking cessation validated by exhaled carbon monoxide in breath (CO-Check, NEOMED GmbH; Germany). Carbon monoxide values > 10 ppm indicate tobacco smoking [42].
- Disease activity
  The recommended EULAR clinical response in relation to disease activity will be assessed using DAS28 at 3 months post-intervention [39]. The DAS28 score consists of four elements; CRP, number of swollen joints, number of painful joints (maximum 28) and a Global General Health VAS score [43, 44]. The joint count and Global General Health are assessed by both the participant and a project nurse blinded to group allocation.

### Secondary outcome measurements

All secondary outcomes are assessed at 3 months (18 weeks post-intervention), 6 months (30 weeks post-intervention) and 12 months follow-up (58 weeks post-intervention).

#### Smoking status

Smoking status will be monitored by self-reported continuous smoking cessation and biochemically validated by exhaled carbon monoxide. Continuous smoking cessation will be measured by asking the patients who report 7-day point prevalence (7 day timeline follow-back) how many days they have not been smoking. Smoking status information is self-reported by the participant and documented in the smoking diary at 3 months post intervention (week 18). To be grouped as having ‘stopped smoking’ both self-reported and biochemical analysis should indicate this.

#### Disease activity

Disease activity will be registered by DAS28, as described above.

#### Flare

Participants will be asked to answer the Flare Instrument, which is a patient self-assessment questionnaire for detecting changes in disease activity among patients with RA [45], designed to detect both past and present disease activity. The questionnaire consists of 12 questions answered on a Likert scale (0 = completely agree, 10 = completely disagree), where higher scores (>2.5) indicate a flare [46]. The Flare Instrument has been translated into Danish and validated [47].

#### Cardiovascular disease risk factors

- Blood pressure (mmHg) and heart rate (beats/min) will be measured after 5 minutes of rest (supine position) (Mobil-O-Graph, IEM; Germany). If it exceeds 140/90 mmHg, two additional measurements will be performed and the mean of the last two will be registered.
- Arterial stiffness will be measured by pulse wave velocity (Mobil-O-Graph, IEM; Germany), where a high value is defined as > 9.9 m/s
- Serum lipids (total cholesterol, high- and low-density lipoprotein cholesterol, triglycerides) and glycated haemoglobin will be measured in venous blood samples
- Waist circumference will be evaluated by a tape measure in centimetres while the patient is standing. For women with a waist circumference > 80 cm there is an increased risk of CVD while the risk is further increased for waist circumference > 88 cm. For men, the measures are defined as > 94 cm and > 102 cm, for increased and further increased risk of CVD, respectively.
- Body weight will be measured to the nearest 0.1 kg (with ordinary clothes, but without shoes) (OBH Nordica, Slim Light, 150 kg, Taastrup, Denmark).
- Height will be measured to the nearest centimetre by a tape measure (without shoes).

#### Lung function

Forced expiratory volume in the first second will be measured with a spirometer (EasyOne^TM^, Model 2001 diagnostic Spirometer, Model 2010 Cradle, NDD Medizintechnik AG; Switzerland).

#### Physical function

HAQ is a standardised questionnaire to assess disability and physical function in patients with RA [48]. The instrument contains 20 items with four possible answers in eight categories, namely dressing, rising from a seat, eating, walking, personal hygiene, stretching for an object, grabbing an object and everyday activities. The questionnaire also includes VAS scales for pain, fatigue and general health. In DANBIO, five additional questions have been added with four possible answers related to physical function, sleep, anxiety and depression. In this study, we will use the 25 questions as used in DANBIO.

#### HR-QoL

HR-QoL is measured using the following two questionnaires:

- SF-36 is a generic instrument measuring HR-QoL by 36 items on eight scales [49]. The scales are physical function, physical activity, limitations, pain, general health, vitality, social function, emotional activity limitations and mental health, and are summarised in two summary scales, namely (1) the physical component scale and (2) the mental component scale.
- EQ-5D is a generic instrument for measuring HR-QoL [50]. The questionnaire contains five items (movement, personal care, usual activities, pain and anxiety/depression) each with five possible ratings.

#### Pain

Pain related to RA is self-reported by participants using the VAS [51]. Participants rate their subjectively experienced level of pain from 0 to 10, where 0 represents ‘no pain’ and 10 represents the ‘worst imaginable pain’. The scale is included in HAQ.

#### Fatigue

The BRAF-NRS assesses fatigue in patients with RA [52]. It includes three questions concerning fatigue (level, effect and coping) over the previous 7 days. Participants rate fatigue on a scale from 0 to 10, where 0 represents ‘no fatigue’ and 10 represents the ‘worst fatigue’.

#### Additional information

We will retrieve data from the DANBIO database regarding the participants’ pharmacological treatment, duration of RA, CRP levels, IgM rheumatoid factor and anti-CCP status. Additional descriptive data include participants’ demography, socioeconomic status, lifestyle (smoking and alcohol) and consumption of pain killers obtained via a questionnaire. Co-morbidities are assessed using the Charlson’s Co-morbidity Scale obtained from the electronic patient journal.

#### Translation

The questionnaires Flare Instrument and BRAF-NRS have been translated from Danish to Norwegian and back translated from Norwegian to Danish [53]. Furthermore, questions regarding demography, socioeconomic status, lifestyle (smoking and alcohol) and consumption of pain killers have been through same translation process as described above.

### Participant timeline

Participants will be followed for 58 weeks, including the 6-week intervention period and a 12 months post-intervention follow-up period.

### Data collection

Data will be collected four times during the trial, namely at baseline and 3, 6 and 12 months post-intervention. All participant-reported questionnaires will be completed electronically on a tablet connected to DANBIO, which will be used only for participants in the trial. Blood samples will be destroyed immediately after the analyses are performed.

### Data management

On each study site participants will receive a trial identification number and all data will be de-identified. The identification list with participant information and trial study number will be kept separate and locked away from the tablet used in the study. De-identified data will be electronically transferred using an online interface via a tablet.

### Statistical analysis

The primary data analysis will be based on the intention-to-treat population. This infers that analyses will be performed on data from all participants disregarding any possible drop-out after randomisation. Missing data among the intention-to-treat population will initially be imputed using the baseline observation carried forward technique [54]. This simplistic ‘null responder imputation’ represents our base case, and is likely valid even if data is ‘missing not at random’ [55] as it assumes and implies that the patients have had no improvement (or worsening) since entering the study (e.g. still smoking with the same disease activity). Furthermore, drop-out analyses will be performed on the patients lost to follow-up.

Continuous outcomes will be analysed using analysis of covariance, adjusting for the variable at baseline and stratifying factors (hospital and anti-CCP status). Categorical data will be analysed using logistic regression; the same covariates will be used for both continuous and categorical data. Statistical significance will be defined as a two-sided *P* value < 0.05, and will follow the logic outlined in this protocol.

Multiple sensitivity analyses will be performed to assess the robustness of the primary analyses, including analyses based on the ‘as observed population’, repeated measures and multiple-imputation analyses, which are all based on model-based approaches for missing data (these details will be available in the final Statistical Analysis Plan).

### Monitoring

#### Ethics, confidentiality and dissemination

The trial will be performed in accordance with the Helsinki Declaration. The project has been approved by The Regional Committee on Health Research Ethics (H-16022001) and the Danish Data Protection Agency (I-suite number 04849). The trial has been reported to Clinicaltrials.gov (NCT02901886). All data and information collected during the trial will be kept confidential and in accordance with the requirements of the Danish and Norwegian Data Protection Agencies and Good Clinical Practice. We plan to publish at least three scientific papers in peer-reviewed journals based on the trial and to disseminate the results to patient organisations and the public through printed and electronic media.

The protocol for this randomised trial is reported in compliance with the Standard Protocol Items: Recommendations for Interventional Trials (SPIRIT) guidelines [56] (Additional file 1).

#### Patient research partner

A patient research partner has been involved in this research trial as recommended by EULAR [57]. We have involved a patient with RA, who is a former smoker. She stopped smoking after being diagnosed with RA and has been involved in the design and final decisions regarding trial outcomes as well as feedback on instruments and questionnaires. The patient research partner has read, commented on and approved the participant information for this trial. Furthermore, she will contribute to the trial with discussion and communication of its results.

#### Access to data

Data will be encrypted and belong to the Center for Rheumatology and Spine Diseases, Rigshospitalet, Glostrup, Denmark. IKR will analyse the data in collaboration with the other authors.

## Discussion

This trial protocol describes the design of an RCT examining the effect of an intensive smoking cessation intervention versus standard care on both smoking cessation and disease activity in smokers with RA. To our knowledge, this is the first RCT in this field.

Current evidence suggests that intensive smoking cessation interventions combining motivational counselling, a teaching programme and NRT achieve the highest long-term smoking cessation rates [22]. Evidence shows that these interventions nearly triple smoking cessation rates compared to smokers attempting to quit without any kind of support [22]. Intensive interventions combining these components also appear the most effective for smokers with chronic diseases [27]. Among smokers with RA there might potentially be a great number of heavy smokers (more than 15 cigarettes per day) [58]. Furthermore, these smokers might have started smoking in their adolescence [58]. There is evidence that motivational interviewing combined with NRT is also effective in heavy smokers and not just in those who smoke less than 15 cigarettes per day. We therefore hypothesise that the intervention in this trial will prove effective for smoking cessation in patients with RA.

Some studies suggest that smokers with RA have a higher need for disease-modifying anti-rheumatic drugs [59, 60]. In addition, they have a poorer response to anti-tumour necrosis factor treatment [61] and have an increased risk of CVD. The mechanisms causing the increased risk are unclear; however, the systemic inflammation and well-known CVD risk factors, such as smoking and high cholesterol levels, could have an influence [8, 62, 63]. All this indicates that smokers with RA could benefit from smoking cessation in regard to their anti-rheumatic medical treatment, which in most patients is lifelong, as well as reducing their risk of CVD events.

This trial will include patients with RA; hence, the study population has the potential to be homogeneous, which increases the reliability between the different study centres. Further strengths of the trial are the randomised design with blinded assessment of the primary outcome disease activity, which limits the risk of detection bias [64]. A potential limitation of the trial is lack of blinding of participants and staff, thus increasing the risk of performance bias. Furthermore, although self-reported smoking cessation is biochemically validated by exhaled carbon monoxide, it should be noted that carbon monoxide levels reach near-normalisation after a 12-hour period of smoking cessation. Participants may therefore self-report smoking cessation and present with normal levels of exhaled carbon monoxide despite having smoked recently [65]. Furthermore, it may be expected that some participants in the control group will be motivated to stop smoking merely due to their participation in the trial; this may potentially limit any incremental effect of the smoking cessation intervention. The included patients’ disease activity will be low or medium and may not achieve a clinically significant change in DAS28. Hence, the risk for type II error will increase.

In conclusion, this trial protocol describes the design of an RCT which aims to examine whether intensive smoking cessation intervention may help smokers with RA to achieve continuous smoking cessation and, secondly, reduce RA disease activity. The trial is clinically important as it aims to generate high quality evidence for the effect of clinical health promotion for smokers with RA. We expect that the results of the trial may be generalisable to other patient groups with chronic inflammatory diseases.

### Trial status

Recruitment for the trial started in October 2016 and is expected to be completed in October 2018.

## Additional file

Additional file 1: SPIRIT 2013 Checklist: recommended items to address in a clinical trial protocol and related documents. (DOC 120 kb)

## Abbreviations

Anti-CCP: anti-cyclic citrullinated peptide
BRAF-NRS: Bristol Rheumatoid Arthritis Fatigue Numeric Rating Scale
CRP: C-reactive protein
DANBIO: Danish National Board of Health Biological Therapies Database
DAS28: Disease Activity Score based on 28 joints
EULAR: The European League Against Rheumatism
HAQ: Health Assessment Questionnaire
HR-QoL: health-related quality of life
NRT: nicotine replacement therapy
RA: rheumatoid arthritis
VAS: Visual analogue scale
